# Comparison of USA and Australian Mobility Device Users’ and Ambulant Bus Users’ Views of Restraints on Public Buses

**DOI:** 10.1093/geroni/igaa057.2337

**Published:** 2020-12-16

**Authors:** Carolyn Unsworth

**Affiliations:** Central Queensland University, Melbourne, Victoria, Australia

## Abstract

Many older people use powered wheelchairs and mobility scooters to access the community on buses but have increased injury risk if the mobility device tips or slides. Wheelchair tie-down and occupant restraint systems (WTORS) are mandated on USA transit buses, and their introduction investigated in Australia. This study examined the views of mobility device and ambulant bus users in the USA and Australia on WTORS. A Qualtrics survey with 448 respondents showed strong support for WTORS use and found the most important factors underpinning use were Safety, Comfort, and Transit time. US research indicates dwell time while fitting WTORS is 4 minutes, and participants reported 5.65(SD3.06) minutes is acceptable. There was no difference in USA and Australian participants who have slid or tipped in their device, despite being restrained in the USA: X2(1,n=220)=.053,p=.53,phi-.016). This research suggests all bus users are supportive of WTORs, but their effectiveness requires investigation.

